# Information Seeking During the Pandemic: The Role of Age, Agency, and Fake News Concerns

**DOI:** 10.1093/geroni/igab046.080

**Published:** 2021-12-17

**Authors:** Ann Pearman, MacKenzie Hughes, Clara Coblenz

**Affiliations:** Georgia Institute of Technology, Atlanta, Georgia, United States

## Abstract

COVID-19 brought rapid changes to the way in which people understand and process news, including both information and misinformation about the pandemic. This cross-sectional study was designed to examine persons’ experiences during the earliest months of the pandemic. The sample included 871 adults ages 20-79 (M=38.27 years, SD=11.40). Online surveys were collected between March and May, 2020 using Amazon Mechanical Turk. Participants completed a series of questionnaires, including a measure of agency from the Midlife Development Inventory, a questionnaire that assessed level of skepticism about the COVID-19 pandemic (i.e. fake news beliefs), a depression scale, a question about their level of anxiety about developing COVID-19, and questions about the frequency in which they sought information about the pandemic from different sources (e.g., TV, social media). A multiple regression using information seeking frequency as the outcome variable revealed several significant relationships. Specifically, younger adults, people with higher agency, and people with higher fake news beliefs all reported higher levels of COVID-19-related information seeking. In addition, there was a significant 3-way interaction between age, agency, and fake news beliefs. Disentangling this interaction revealed that older adults with low agency were least likely to engage in information seeking. There were, however, no age differences in information seeking in participants with high agency and fake news beliefs, but large age differences in participants with low agency but high fake news beliefs. Findings suggest agency is an important predictor of information seeking behavior, particularly for older adults with high levels of skepticism about the pandemic.

